# A novel validated method for predicting the risk of re-hospitalization for worsening heart failure and the effectiveness of the diuretic upgrading therapy with tolvaptan

**DOI:** 10.1371/journal.pone.0207481

**Published:** 2018-11-14

**Authors:** Hideyuki Takimura, Tasuku Hada, Mami Kawano, Takayuki Yabe, Yukako Takimura, Satoru Nishio, Masatsugu Nakano, Reiko Tsukahara, Toshiya Muramatsu

**Affiliations:** Department of Cardiology, Tokyo General Hospital, Tokyo, Japan; Universita degli Studi di Napoli Federico II, ITALY

## Abstract

Increased re-hospitalization due to acute decompensated heart failure (ADHF) is a modern issue in cardiology. The aim of this study was to investigate risk factors for re-hospitalization due to worsening heart failure, and the effect of tolvaptan (TLV) on decreasing the number of re-hospitalizations. This was a multicenter, retrospective study. The re-hospitalization factors for 1191 patients with ADHF were investigated; patients receiving continuous administration of TLV when they were discharged from the hospital (n = 194) were analyzed separately. Patients were classified into 5 risk groups based on their calculated Preventing Re-hospitalization with TOLvaptan (Pretol) score. The total number of patients re-hospitalized due to worsening heart failure up to one year after discharge from the hospital was 285 (23.9%). Age ≥80 years, duration since discharge from the hospital after previous heart failure <6 months, diabetes mellitus, hemoglobin <10 g/dl, uric acid >7.2 mg/dl, left ventricular ejection fraction (LVEF) <40%, left atrial volume index (LAVI) >44.7 ml/m2, loop diuretic dose ≥20 mg/day, hematocrit <31.6%, and estimated glomerular filtration rate (eGFR) <50 ml/min/1.73m2 were independent risk factors for re-hospitalization for worsening heart failure. There was a significant reduction in the re-hospitalization rate among TLV treated patients in the Risk 3 group and above. In conclusions, age, duration since previous heart failure, diabetes mellitus, hemoglobin, uric acid, LVEF, LAVI, loop diuretic dose, hematocrit, and eGFR were all independent risk factors for re-hospitalization for worsening heart failure. Long-term administration of TLV significantly decreases the rate of re-hospitalization for worsening heart failure in patients with a Pretol score of 7.

## Introduction

Re-hospitalization due to worsening heart failure has become a serious issue in modern cardiology. Factors contributing to total death and cardiovascular death have been studied in many large registries in Japan [1,2]. In the Japanese Cardiac Registry of Heart Failure in Cardiology (JCARE-CARD), the rate of re-hospitalization due to worsening heart failure was 27% within 6 months of discharge from the hospital, and 35% after one year [3]. Additionally, 36.2% of participants in the ATTEND registry had a history of hospital treatment for heart failure [2]. The rate of re-hospitalization for heart failure is high. Previous studies showed that angiotensin-converting enzyme inhibitors (ACE-I), angiotensin II receptor blockers (ARB) [4–7], and β blockers (BB) [8–11] reduce heart failure deaths and improve patient prognosis. However, while there has been a decrease in deaths due to the administration of these drugs, the rate of re-hospitalization due to heart failure has not been reduced [12].

Volume overload is the leading cause of and therapeutic target for worsening heart failure [13, 14]. Diuretics are administered to patients with the goal of fluid control, but loop diuretics, mainly furosemide, may worsen patient prognosis [15, 16]. Tolvaptan (TLV) is an oral selective vasopressin-2 receptor antagonist and a diuretic. The short-term efficacy of TLV was verified in the Efficacy of Vasopressin Antagonism in Heart Failure Outcome Study With Tolvaptan (EVEREST) trial, but the long-term efficacy was found to be neutral [17]. Nevertheless, the rate of re-hospitalization was decreased in patients in the TLV treatment group with both heart failure and chronic kidney disease (CKD) [18]. However, it is not yet clear which symptoms, other than CKD, correlate with a decreased re-hospitalization rate due to TLV treatment. Thus, this study aimed to identify heart failure related re-hospitalization factors, and to determine the profile of patients for whom it is possible to decrease the re-hospitalization rate by upgrading from conventional diuretics to TLV.

## Methods

### Study population

**Fig 1:**
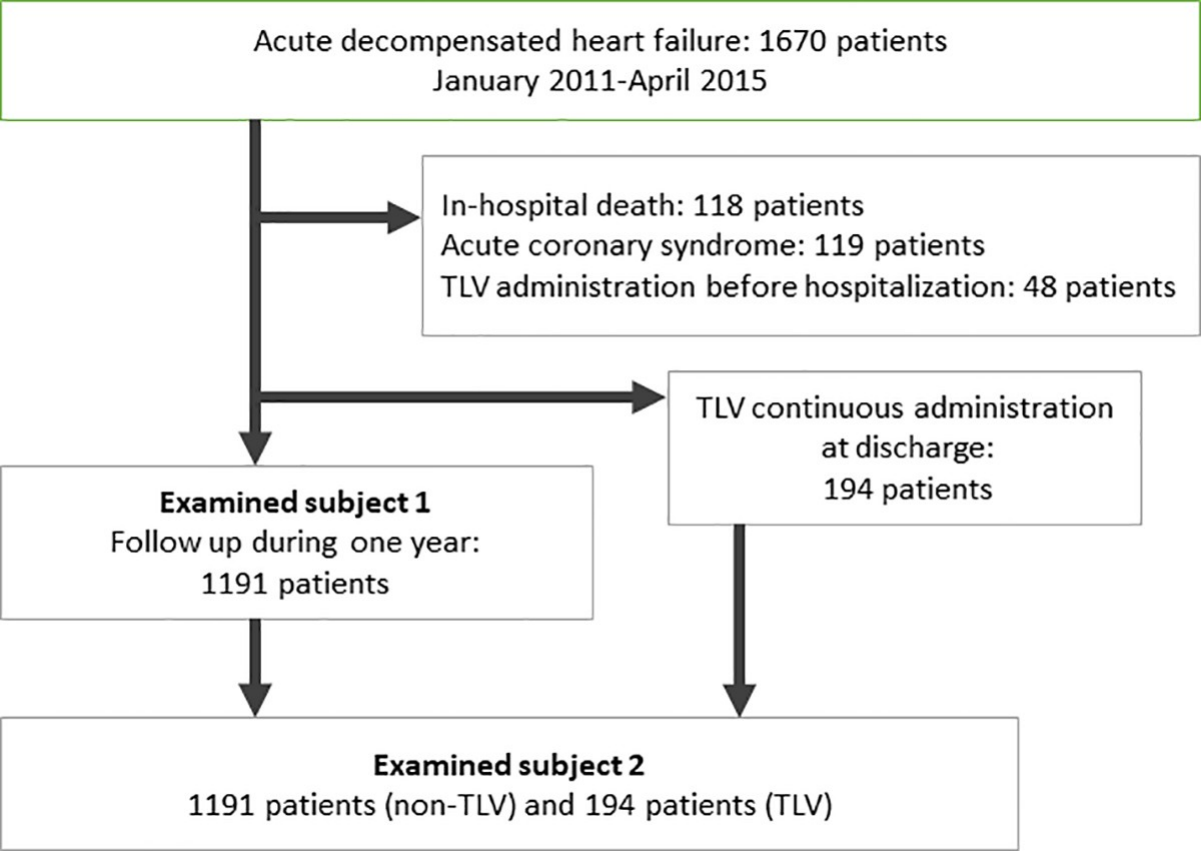
Patient flow chart of the present study. Examined subject 1 included a total of 1191 patients to investigate the risk heart failure-related re-hospitalization. Examined subject 2 investigated the effect of continuous administration of Tolvaptan (TLV), and included the 1191 patients from examined subject 1, as well as the patients who received continuous administration of TLV when they were discharged from the hospital (n = 194).

This was a multicenter, retrospective study (January 2011-December 2016) of 1670 patients hospitalized for acute decompensated heart failure (ADHF). Patients with acute coronary syndrome (n = 119), cases of in-hospital death (n = 118), patients who were administered TLV before hospitalization (n = 48) and patients who were implanted left ventricular assist device as destination therapy were excluded from the study population. Examined subject 1 excluded patients who received continuous administration of TLV when they were discharged from the hospital, resulting in inclusion of 1191 patients, to investigate the risk of heart failure-related re-hospitalization. Examined subject 2 studied the effect of continuous administration of TLV, and included the 1191 patients from Examined subject 1 as well as patients who received continuous administration of TLV when they were discharged from the hospital (n = 194) (Fig 1). A follow-up study was conducted to determine whether or not patients were re-hospitalized for worsening heart failure within a year of discharge from the hospital. The attending physician diagnosed ADHF based on the Framingham criteria [19]. All patients were symptomatic, with New York Heart Association (NYHA) classifications of II-IV. They received treatment based on heart failure guidelines. TLV was continuously administered to patients at the time of discharge from the hospital if the patient's condition had worsened while already on a diuretic after acute stage fluid control. The protocol of this study conforms to the Declaration of Helsinki, and was approved by the ethics committee of the Tokyo General hospital. Informed consent was obtained from all patients.

### Data collection

Data for the following demographic and clinical variables were collected during the period of hospitalization: sex, age, past smoking, current smoking, disease complications, and etiology. Any past history of hospitalization for heart failure and the period of time between discharge from the hospital and the current re-hospitalization were investigated. Chronic obstructive pulmonary disease was defined as a history of diagnosis of or treatment for it. Hypertension was defined as a history of diagnosis of or treatment for hypertension. Diabetes mellitus was defined as meeting the World Health Organization criteria for diabetes, or receiving treatment for diabetes. History of ischemic heart disease was defined as previous pharmaceutical treatment for ischemic heart disease or a history of revascularization. Serum creatinine levels when patients were admitted versus discharged from the hospital were compared, and patients with elevated levels upon discharge were classified as "elevated creatinine at discharge." Worsening renal function was defined as a serum creatinine elevation of 0.3 mg/dL or 50% above baseline within 48 h [20]. Heart failure with reduced ejection fraction (HFrEF) was defined as a left ventricular ejection fraction (LVEF) ≤40%. Blood tests and echocardiography were conducted within one week before discharge from the hospital. Estimated glomerular filtration rate (eGFR) was calculated based on the Modification of Diet in Renal Disease study equation coefficients (Japanese version). CKD was defined as an eGFR <60 ml/min/1.73m2. Left ventricular end systolic and diastolic diameter (LVDd and LVDs), left ventricular end systolic and diastolic volume (ESV and EDV), stroke volume (SV), left ventricular ejection fraction (LVEF), left atrial diameter (LAD), and left atrial volume index (LAVI) were measured by echocardiography. Mitral regurgitation was defined as moderate or higher levels of mitral regurgitation. The following pharmacological treatments at the time of discharge were investigated: ACE-I, ARB, BB, aldosterone antagonists, and loop diuretics. This study investigates the rate of re-hospitalization due to worsening heart failure within one year after hospital discharge.

### Statistical analysis

All continuous data are presented as either mean ± standard deviation or median (interquartile range). The unpaired Student’s t test was used for comparisons of continuous variables between two groups. If the data were not normally distributed, the Mann-Whitney U test was used. Categorical variables are presented as frequency and percentage. The Chi-square test or Fisher's exact test was used for comparisons of proportions. A multiple Cox regression model analysis was conducted to determine re-hospitalization factors during the one-year period. Prominent clinically relevant factors with a p value ≤0.10 on univariate analysis were included in the multivariate analysis model. Significant factors with a p value <0.05 were included or not included based on backward stepwise selection. Additionally, a model of significant diuretic-related re-hospitalization factors was created. This model included only the main effect of factors, and interactions were not included. The calibration of the model was evaluated using a corresponding plot. A simple risk scoring system was created using the significant independent re-hospitalization factors. Points were assigned, for a total of 15 points, in order to correlate the score with the hazard ratio determined by the multiple Cox regression model. Patients were classified into risk severity groups based on their scores. A comparison of the rate of re-hospitalization for worsening heart failure was conducted using the log-rank test for Kaplan-Meier curves. A p-value of <0.05 was considered statistically significant. Statistical analysis was performed using JMP 12 (SAS Institute, Cary, NC, USA) and SPSS (SPSS Inc., Chicago, IL, USA).

## Results

### Baseline characteristics

Out of 1191 patients, the total number of patients who were re-hospitalized for worsening heart failure within a year of discharge from the hospital was 285 (23.9%). The study population was split into a re-hospitalization group (n = 285) and a no re-hospitalization group (n = 906) for comparative analysis. There were no significant differences in the following demographic and clinical variables: sex, past or current smoking status, presence of atrial fibrillation, or the incidence of worsening renal function. The age in the re-hospitalization group was significantly higher (81.1±11.1 vs. 77.9±12.6 years, p<0.001). Significantly more patients in the re-hospitalization group had a history of heart failure, and those patients also had a shorter period of time between discharge from the previous hospitalization and the present re-hospitalization. There were significantly more patients with CKD, hypertension, diabetes mellitus, ischemic heart disease, and HFrEF in the re-hospitalization group. The re-hospitalization group had a significantly shorter period of stay in the hospital. The laboratory variables at discharge in the re-hospitalization group indicated significantly lower levels of hemoglobin, hematocrit, and eGFR, as well as significantly higher uric acid and blood urea nitrogen (BUN) levels. There were no differences between groups in terms of albumin, serum creatinine, sodium, potassium, or N-terminal pro b-type natriuretic peptides (NT-pro BNP) levels. The echocardiography variables at discharge in the re-hospitalization group indicated significantly larger LVDd, LVDs, EDV, ESV, and LAVI, as well as significantly lower SV and LVEF. There was no difference between groups in the incidence of moderate or more severe mitral regurgitation. The pharmacological treatment at discharge included significantly higher proportions of β-blockers and diuretics in the re-hospitalization group. The re-hospitalization group received significantly larger doses of loop diuretics (22.3±15.9 versus 19.1±21.0 mg, p = 0.008). There were no differences between groups in the proportion of patients who were administered ACE-I, ARB, or aldosterone antagonists (Table 1).

**Table 1:** Characteristics of patients.

	Re-hospitalization (n = 285)	No re-hospitalization (n = 906)	p value
**Demographic and clinical variables**			
**Male, n**	158(55.4%)	534(58.9%)	0.30
**Age, years**	81.1±11.1	77.9±12.6	<0.001
**Prior history of heart failure hospitalization, n**	117(41.1%)	170(18.8%)	<0.001
**Duration since previous heart failure at discharge, days**	153.2±192.6	242.2±299.2	0.004
** <3 months, n**	64(22.5%)	68(7.5%)	<0.001
** <6 months, n**	84(29.5%)	89(9.8%)	<0.001
**Past smoking, n**	109(38.3%)	338(37.3%)	0.87
**Current smoking, n**	41(14.4%)	143(15.8%)	0.60
**Chronic obstructive pulmonary disease**	25(8.8%)	63(7.0%)	0.31
**Chronic kidney disease, n**	215(75.4%)	605(66.8%)	0.006
**Hypertension, n**	161(56.5%)	443(48.9%)	0.025
**Diabetes mellitus, n**	110(38.6%)	247(27.3%)	<0.001
**History of ischemic heart disease, n**	68(23.9%)	153(16.9%)	0.008
**Atrial fibrillation, n**	25(8.8%)	102(11.3%)	0.29
**HFrEF, n**	123(43.5%)	267(29.5%)	<0.001
**Length of hospitalization, days**	17.6±12.7	20.6±19.0	0.003
**Length of hospitalization>30days, n**	33(11.6%)	164(18.1%)	0.01
**Elevated creatinine at discharge, n**	37(13%)	104(11.5%)	0.33
**Worsening renal function, n**	162(34.6%)	49(35.3%)	0.84
**Etiology**			
**Arrhythmia, n**	15(5.3%)	63(7.0%)	0.003
**Congenital heart disease, n**	3(1.1%)	4(0.4%)	
**Ischemic cardiomyopathy, n**	106(37.2%)	256(28.3%)	
**Pulmonary hypertension, n**	3(1.1%)	13(1.4%)	
**Valvular disease, n**	67(23.5%)	177(19.5%)	
**Cardiomyopathy, n**	78(27.4%)	286(31.6%)	
**Others, n**	13(4.6%)	107(11.8%)	
**Laboratory variables at discharge**			
**Hemoglobin, g/dl**	11.2±1.9	11.7±2.2	<0.001
**Hematocrit, %**	33.9±5.6	35.5±6.3	<0.001
**Albumin, g/dl**	3.1±0.4	3.2±0.5	0.20
**Uric acid, mg/dl**	7.5±2.1	7.1±2.2	0.01
**Urea nitrogen, mg/dl**	27.5±14.5	25.2±15.8	0.03
**Creatinine, mg/dl**	1.4±0.8	1.5±1.6	0.53
**eGFR, ml/min/1.73m2**	43.7±24.2	49.2±25.0	0.002
**Sodium, mEq/l**	139.1±3.7	139.2±3.8	0.70
**Potassium, mEq/l**	4.2±0.6	4.2±0.6	0.67
**NT-pro BNP, pg/ml**	7059.5±8777.1	5780.3±13950.9	0.092
**Echocardiographic variables at discharge**			
**LVDd, mm**	51.7±10.0	49.3±8.9	<0.001
**LVDs, mm**	40.8±11.7	37.5±10.3	<0.001
**EDV, ml**	110.7±59.1	101.8±50.1	0.026
**ESV, ml**	68.4±50.4	57.6±40.9	0.001
**SV, ml**	42.3±16.1	44.3±18.6	0.046
**LVEF, %**	43.0±14.2	47.7±14.6	<0.001
**Mitral regurgitation, n**	34(11.9%)	100(11.4%)	0.77
**LAD, mm**	42.3±7.4	41.4±8.1	0.12
**LAVI, ml/m2**	46.2±19.2	40.8±24.7	0.004
**ICD, CRT**	42(14.7%)	33(3.6%)	<0.001
**Pharmacological treatment at discharge**			
**ACE-I or ARB, n**	183(64.2%)	582(64.3%)	0.81
**β-blocker, n**	199(69.8%)	554(61.2%)	0.008
**Aldosterone antagonists, n**	100(35.1%)	363(40.1%)	0.078
**Loop diuretics, n**	231(81.1%)	631(69.7%)	<0.001
**Dose of loop diuretics, mg**	22.3±15.9	19.1±21.0	0.008

Values are mean±SD, %, or median (quartile 1–quartile 4).

eGFR = estimated glomerular filtration rate; NT-pro BNP = N-terminal pro-brain natriuretic peptide; LVDd and LVDs = left ventricular end systolic and diastolic diameter; ESV and EDV = left ventricular end systolic and diastolic volume; SV = stroke volume; LVEF = left ventricular ejection fraction; LAD = left atrial diameter; LAVI = left atrial volume index; ICD = Implantable Cardioverter Defibrillators; CRT = Cardiac Resynchronization Therapy; ACE = angiotensin-converting enzyme; ARB = angiotensin receptor blocker.

### Re-hospitalization factors for worsening heart failure

**Fig 2:**
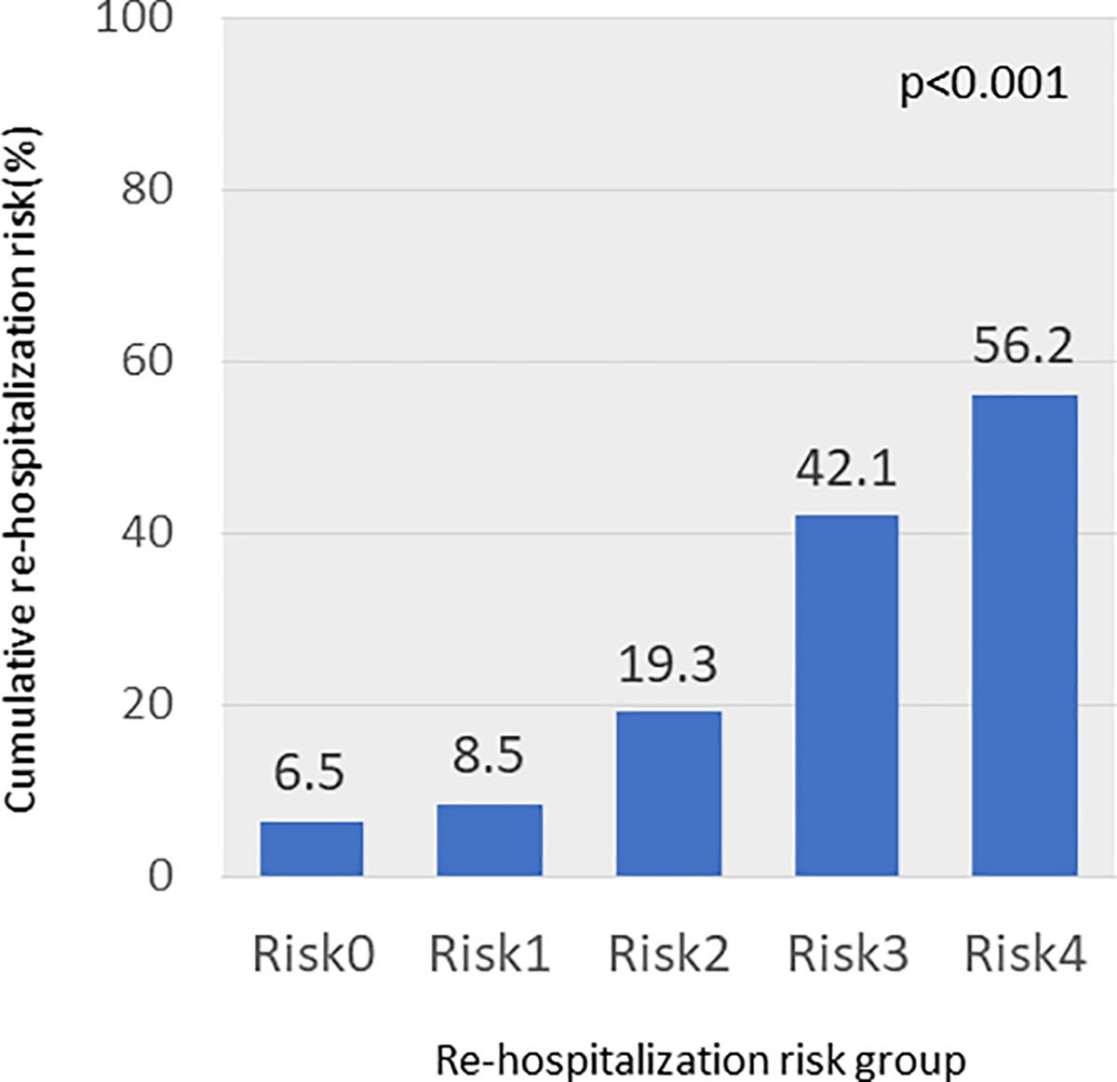
Cumulative re-hospitalization risk. Rates of re-hospitalization for congestive heart failure in the validation subset based on their risk score.

**Fig 3:**
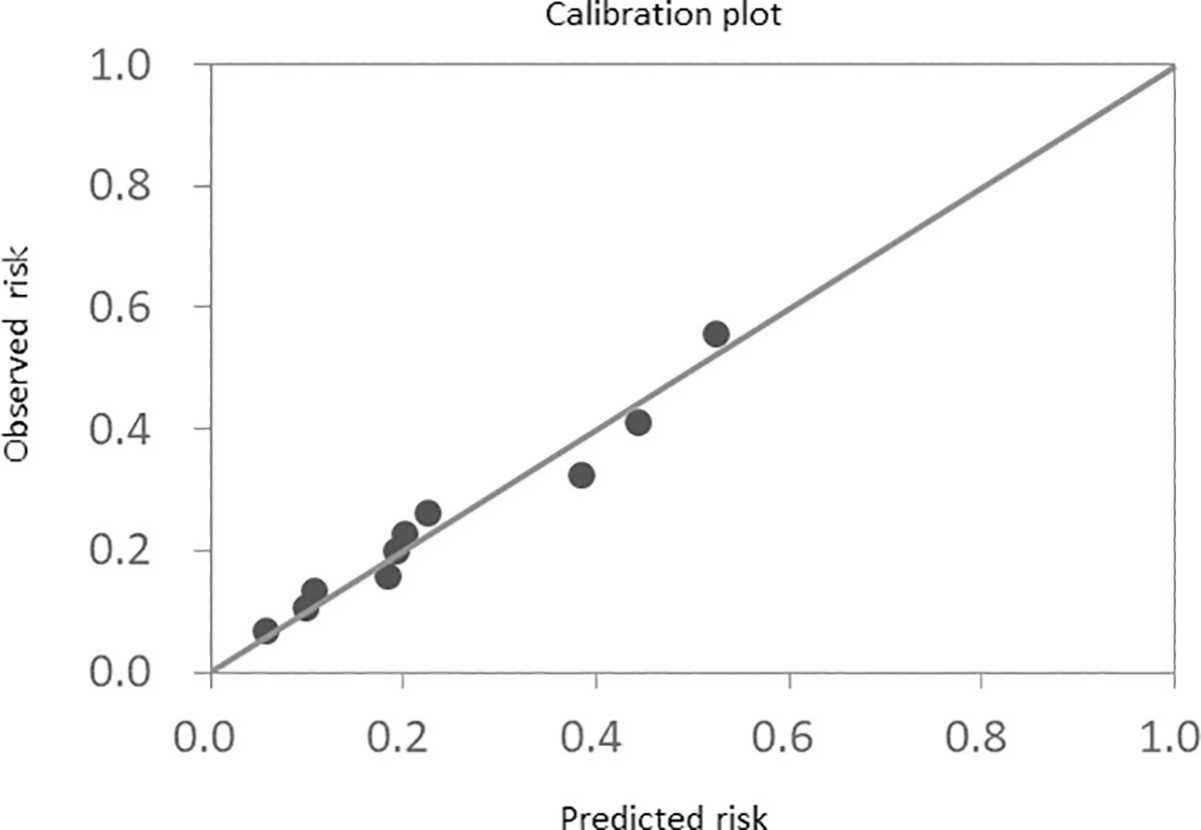
Calibration plot. Calibration plot of the 1-year hospitalization model for worsening of heart failure (external validation).

The results of multivariate analysis showed that age ≥80 years (hazard ratio (HR) 1.87, 95% confidence interval (CI) 1.38–2.55, p<0.001), amount of time since the previous discharge from the hospital <6 months (HR 3.04, 95% CI 2.14–4.32, p<0.001), diabetes mellitus (HR 1.77, 95% CI 1.30–2.41, p<0.001), hemoglobin <10 g/dl (HR 1.47, 95% CI 1.07–2.03, p = 0.02), uric acid >7.2 mg/dl (HR 1.54, 95% CI 1.14–2.09, p = 0.005), LVEF <40% (HR 1.93, 95% CI 1.44–2.61, p<0.001), and LAVI >44.7 ml/m2 (HR 1.61, 95% CI 1.16–2.25, p = 0.005), were all independent factors. Additionally, an analysis (multivariate model 2) of diuretic-related re-hospitalization factors was conducted in order to compare the efficacy of these factors with the efficacy of TLV, which is also a diuretic. Independent factors for re-hospitalization were: loop diuretic dose ≥20 mg/day (HR 1.55, 95% CI 1.13–2.16, p = 0.007), hematocrit <31.6% (HR 1.58, 95% CI 1.17–2.13, p = 0.003), and eGFR <50 ml/min/1.73m2 (HR 1.69, 95% CI 1.19–2.42, p = 0.003) (Table 2). We created a 15-point scale based on the results of the multivariate analysis to create a standardized score for risks of re-hospitalization due to heart failure (Table 3). We classified patients based on their score on this scale as follows: Risk 0 (score 0, 2.6%), Risk 1 (score 1–3, 23.9%), Risk 2 (score 4–6, 43.1%), Risk 3 (score 7–9, 24.4%), and Risk 4 (score ≥0, 6.1%). The cumulative re-hospitalization risk of each group was 6.5%, 8.5%, 19.3%, 42.1%, and 56.2%, respectively (p<0.001) (Fig 2). The re-hospitalization risk rose as the cumulative re-hospitalization risk rose. The AUC for this model was 0.72. The external validation of the calibration ability of this model is shown in the calibration plot in Fig 3. The p-value for the Hosmer-Lemeshow test was not significant.

**Table 2:** Univariable and multivariable predictors of 1-year readmission for worsening of heart failure.

	Univariable			Multivariate model 1			Multivariate model 2		
	HR	95%CI	p value	HR	95%CI	p value	HR	95%CI	p value
Age≥80years	1.69	1.28–2.24	<0.001	1.87	1.38–2.55	<0.001			
Length of hospitalization>30days	0.59	0.39–0.87	0.008						
Prior history of heart failure hospitalization	3.02	2.26–4.03	<0.001						
Duration since previous heart failure at discharge(<6months)	3.84	2.74–5.37	<0.001	3.04	2.14–4.32	<0.001			
Chronic kidney disease	1.53	1.13–2.08	0.005						
Hypertension	1.36	1.04–1.78	0.025						
Diabetes mellitus	1.68	1.27–2.22	<0.001	1.77	1.30–2.41	<0.001			
History of ischemic heart disease	1.54	1.11–2.12	0.001						
Loop diuretics	1.86	1.35–2.61	<0.001						
Dose of loop diuretics≥20mg	1.8	1.34–2.46	<0.001				1.55	1.13–2.16	0.007
Hemoglobin<10g/dl	1.65	1.22–2.22	0.001	1.47	1.07–2.03	0.02			
Hematocrit<31.6%	1.68	1.26–2.23	<0.001				1.58	1.17–2.13	0.003
Uric acid>7.2mg/dl	1.86	1.40–2.47	<0.001	1.54	1.14–2.09	0.005			
Urea nitrogen>17.7mg/dl	1.51	1.14–1.99	0.004						
eGFR<50ml/min/1.73m2	1.9	1.36–2.71	<0.001				1.69	1.19–2.42	0.003
LVDd>55mm	1.73	1.29–2.32	<0.001						
LVDs>47.2mm	2.14	1.56–2.91	<0.001						
EDV>161ml	1.84	1.27–2.64	0.001						
ESV>117ml	2.47	1.66–3.63	<0.001						
LVEF<40%	1.82	1.38–2.39	<0.001	1.93	1.44–2.61	<0.001			
LAVI>44.7ml/m2	1.79	1.32–2.42	<0.001	1.61	1.16–2.25	0.005			

**Table 3:** The Pretol score.

Re-hospitalization risk	HR	Adjustment factor points
Hemoglobin<10g/dl	1.47	1
Uric acid>7.2mg/dl	1.54	1
Dose of loop diuretics≥20mg	1.55	1
Hematocrit<31.6%	1.58	1
LAVI>44.7	1.61	1
eGFR<50	1.69	1
Diabetes mellitus	1.77	2
Age≥80years	1.87	2
LVEF<40	1.93	2
Duration since previous heart failure at discharge(<6months)	3.04	3
**Total score**		15 points
**Risk group**	**Risk score, points**	**Patients, n(%)**
Risk 0	0	31(2.6%)
Risk 1	1–3	284(23.9%)
Risk 2	4–6	513(43.1%)
Risk 3	7–9	290(24.4%)
Risk 4	10<	73(6.1%)

### Re-hospitalization rate reduced by continuous administration of TLV

**Fig 4:**
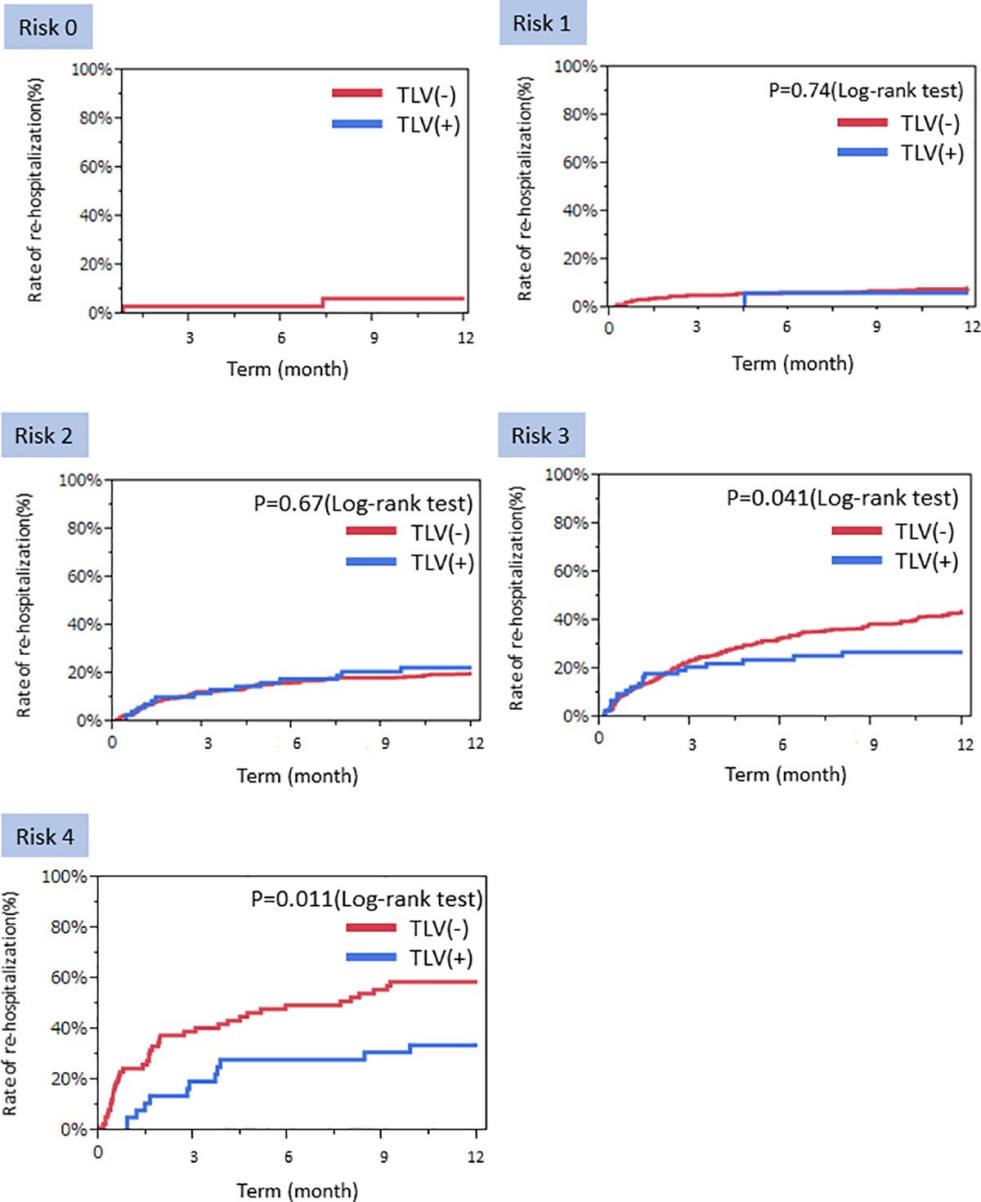
Cumulative incidence curves of re-hospitalizations risk. Cumulative incidence curves of re-hospitalizations risk for worsening of heart failure after 1-year follow-up in the validation subset based on their risk score. Comparison of TLV versus no TLV.

A comparative analysis between patients included in Examined subject 1 (n = 1191) and patients who received continuous administration of TLV when they were discharged (n = 194) was conducted in order to investigate the reduction of the rate of re-hospitalization due to continuous administration of TLV (S1 Table). The reduction was higher in the TLV(+) group than in the TLV(-) group for past history of hospitalization for heart failure, CKD, and HFrEF. In terms of blood tests at the time of discharge, hemoglobin, hematocrit, and eGFR were significantly lower in the TLV(+) group compared to the TLV(-) group. Uric acid values, blood urea nitrogen (BUN), and NT-pro BNP were significantly higher in the TLV(+) group. LVDd, LVDs, EDV, ESV, and LAVI were significantly higher in the TLV(+) group, and LVEF was significantly smaller in the TLV(+) group. The proportions of β-blockers and diuretics used in the TLV(+) group were significantly higher. Loop diuretics were administered to every patient in the TLV(+) group. The doses of loop diuretics were significantly higher in the TLV(+) group. There were no differences in the proportions of ACE-I, ARB, and aldosterone antagonists used between groups. The average continuous dose of TLV was 9.3±4.9 mg. The re-hospitalization risk groups within the TLV(+) group were as follows: Risk 0 (n = 0), Risk 1 (16, 8.3%), Risk 2 (68, 35.1%), Risk 3 (72, 37.1%), Risk 4 (19.6%). S1 Table shows comparisons between each of the risk groups in the TLV(-) and TLV(+) groups. Log-rank tests for Kaplan-Meier curves were used to investigate the rate of re-hospitalization due to worsening heart failure within one year amongst the risk groups (Fig 4). The re-hospitalization rate for the Risk 0 group after one year was 6.5%. For the Risk 1 group, the re-hospitalization rate after one year was 8.6% in the TLV(-) group and 6.3% in the TLV(+) group; there was no significant difference between the TLV(-) and TLV(+) groups. In the Risk 2 group, re-hospitalization rates were 20.1% and 22.5%, respectively, with no significant difference. In contrast, the Risk 3 group re-hospitalization rates were 43.8% and 27.0%; the rate in the TLV(+) group was significantly lower (p = 0.041) than that of the TLV(-) groups. Similarly, in Risk 4 group, the re-hospitalization rates were significantly lower for the TLV(+) group (33.9%) compared to the TLV(-) group (58.8%) (p = 0.011).

## Discussion

In this study, we calculated a score to predict re-hospitalization for worsening heart failure, and showed the efficacy of TLV treatment for decreasing the rate of re-hospitalization. The main outcomes of this study were as follows. First, we created a simple scoring system to predict the likelihood of re-hospitalization that can be calculated using 10 factors in everyday clinical practice. Second, we investigated the efficacy of TLV for reducing the rate of re-hospitalization in different risk groups based on the predictive scores using re-hospitalization factors. As a result, we demonstrated the efficacy of TLV for reducing the re-hospitalization rate in Risk group 3 and higher. Thus, if this score (Preventing Re-hospitalization with TOLvaptan score, Pretol) is 7 or higher, TLV can be expected to reduce the risk of re-hospitalization. This is the first clear definition of specific patient profiles in which sustained TLV administration is effective.

### Regarding the re-hospitalization for worsening heart failure risk factor score (Pretol score)

In this study, the total number of patients who were re-hospitalized for worsening heart failure within one year of discharge was 285 (23.9%). This is consistent with the re-hospitalization rate from research conducted in Japan in the JCARE-CARD study [1]. As shown by the Pretol score in the current study, age ≥80 years, duration since discharge from the hospital <6 months, diabetes mellitus, hemoglobin <10 g/dl, uric acid >7.2 mg/dl, LVEF <40%, LAVI >44.7 ml/m2, loop diuretic dose ≥20 mg/day, hematocrit <31.6%, and eGFR <50 ml/min/1.73m2 are all independent risk factors for re-hospitalization for worsening heart failure. Previous research using the Redin-SCORE, which predicts the risk of re-hospitalization for worsening heart failure, shows that the presence of Framingham left HF signs, eGFR <60 mL/min/m2, BNP >43 pmol/L (>150 ng/L) or NT-pro BNP >118 pmol/L (>1000 ng/L), heart rate >70 bpm, the presence of anemia, and a left atrial size >26 mm/m2 are risk factors for re-hospitalization for worsening heart failure [21]. The C-statistic in the Redin-SCORE model is 0.67, but the C-statistic for the model presented in the current study is higher, at 0.72. Additionally, other research indicates that age ≥75 years, diabetes mellitus, ICM, diastolic BP at discharge ≥70 mmHg, heart rate at discharge ≥75 bpm, and use of loop diuretics are all risk factors for re-hospitalization for worsening heart failure [22]. A previous study reported that emphysema, NYHA class, potassium, SBP, physical activity in patients who are 65 years of age or older, CKD, NYHA class, SBP in patients who are younger than 65 years of age, previous HF hospitalization, coronary artery disease, CKD, emphysema, alcohol consumption, smoking, NYHA class, and systolic blood pressure (SBP) in patients who are 75 years of age or older are all risk factors for re-hospitalization [23]. A simple risk score to predict the risk of re-hospitalization has been reported previously, but the C-statistic was 0.60. The Pretol score used in the current study had a higher C-statistic of 0.72 for the prediction of re-hospitalization for worsening heart failure. As shown in Fig 3, a calibration plot can be used to predict the chance of re-hospitalization for worsening heart failure. This is because we conducted an investigation of diuretic-related re-hospitalization factors. The correlation between the dose of loop diuretics and prognosis has been reported previously; the larger the dose of loop diuretics, the poorer the prognosis [24]. Also, the administration of diuretics causes changes in hematocrit and GFR. Accordingly, these factors correlate strongly with re-hospitalization risk. Conventional scores for predicting the risk of re-hospitalization for worsening heart failure could not predict the efficacy of TLV. This is because they did not include factors affected by diuretics in the analyses. This is the major point of improvement made by the scoring system proposed in this study.

### The efficacy of TLV on reducing re-hospitalization rates

In this study, we performed risk stratification based on risk factors for re-hospitalization for worsening heart failure, and investigated the efficacy of continuous TLV administration in reducing the rate of re-hospitalization after one year. As a result, we showed that the continuous administration of TLV decreased the re-hospitalization rate by 17% in Risk group 3 and by 25% in Risk group 4. There are few studies reporting that chronic treatment with heart failure medications lead to a decrease in re-hospitalization for worsening heart failure. However, the current analysis demonstrates the efficacy of TLV against chronic heart failure.

TLV is a new antidiuretic that inhibits the vasopressin V2 receptor in the renal collecting duct. Its mechanism of action differs from existing diuretics; it is an aquaretic that does not bring about changes in kidney circulation, changes in electrolytes, or changes in hemodynamics [17]. The EVEREST study reported that TLV decreased urinary volume and body weight, as well as reduced swelling [17]. When used in response to ADHF, the urinary volume and the incidence of worsening renal function decreased significantly 48 hours after initiation of administration when compared to existing diuretics [25]. Additionally, joint administration of TLV and carperitide in response to ADHF has little effect on hemodynamics, and it shortens the length of time for hyperemia improvement [26]. The EVEREST trial demonstrated improvement in heart failure symptoms, but there was no significant difference in prognosis [17]. However, concerning long-term treatment with TLV in patients that also have CKD, the rate of re-hospitalization within 6 months in patients whose renal function did not worsen was 61.1% in the loop diuretic group and 36.4% in the TLV group, indicating a significant reduction in the re-hospitalization rate with TLV prescription [18]. Also, with regards to kidney function, the K-STAR study compared the effect of an increased dose of loop diuretics with the addition of TLV administration at the time of worsening heart failure in patients with CKD; results of this study showed that the TLV treatment group had a lower risk of worsening renal function [27]. Thus, TLV is different from loop diuretics in that it protects against worsening renal function. The current study showed a significant reduction in re-hospitalization for worsening heart failure with TLV use in patients with a Pretol score of 7 or higher. The efficacy of TLV in reducing the risk of re-hospitalization in patients with CKD as well as heart failure has been proven, but this study also demonstrates the patient profiles for which TLV can be expected to reduce the risk of re-hospitalization. This suggests that long-term administration of TLV may reduce the risk of re-hospitalization for worsening heart failure in patients with a Pretol score of 7 or higher.

### Study limitations

This study has several limitations. The first is that although it is a multicenter retrospective study, it is not a randomized, double-blind, placebo-controlled study. Thus, there was a significant difference between baseline patient background factors in the two groups. The second is that the number of patients receiving continuous administration of TLV was smaller than the number of patients who did not receive continuous TLV administration. Also, TLV administration was determined at the discretion of the doctors in charge of the administration standards. Thus, in order to conduct a comparison taking into account patient background, the rate of re-hospitalization was compared for different risk levels. The third limitation is that re-hospitalization risk factors, such as Framingham left HF signs and heart rate [21], 6 minutes walking test [28] and physical activity [29] or frailty [30] could not be investigated.

## Conclusion

In patients with heart failure, age ≥80 years, duration since discharge from the hospital <6 months, diabetes mellitus, hemoglobin <10 g/dl, uric acid >7.2 mg/dl, LVEF <40%, LAVI >44.7 ml/m2, loop diuretic dose ≥20 mg/day, hematocrit <31.6%, and eGFR <50 ml/min/1.73m2 are all independent risk factors for re-hospitalization for worsening heart failure. Additionally, this study suggests that the long-term administration of TLV may cause a significant reduction in the rate of re-hospitalization for worsening heart failure in patients with a Pretol score of 7 or more calculated based on these factors.

## Supporting information

S1 Table Characteristics of patients for each risk group. Values are mean±SD, %, or median (quartile 1–quartile 4). *Significant p value.
(XLSX)
